# Spatial Multicriteria Evaluation for Mapping the Risk of Occurrence of Peste des Petits Ruminants in Eastern Africa and the Union of the Comoros

**DOI:** 10.3389/fvets.2019.00455

**Published:** 2019-12-12

**Authors:** Anne-Sophie Ruget, Annelise Tran, Agnès Waret-Szkuta, Youssouf Ousseni Moutroifi, Onzade Charafouddine, Eric Cardinale, Catherine Cêtre-Sossah, Véronique Chevalier

**Affiliations:** ^1^UMR ASTRE, CIRAD, Ste-Clotilde, France; ^2^ASTRE, Univ Montpellier, CIRAD, INRAE, Montpellier, France; ^3^UMR TETIS, CIRAD, Ste-Clotilde, France; ^4^TETIS, Univ Montpellier, AgroParisTech, CIRAD, CNRS, INRAE, Montpellier, France; ^5^IHAP, Université de Toulouse, INRAE, ENVT, Toulouse, France; ^6^Ministry of Agriculture, Fisheries, Environment, Territorial Development, and Urbanism, Moroni, Comoros; ^7^Epidemiology and Public Health Unit, Institut Pasteur du Cambodge, Phnom Penh, Cambodia

**Keywords:** geographic information system, multi-criteria evaluation, peste des petits ruminants, Eastern Africa, Union of the Comoros, risk mapping

## Abstract

Peste des petits ruminants virus (PPRV), responsible for peste des petits ruminants (PPR), is widely circulating in Africa and Asia. The disease is a huge burden for the economy and development of the affected countries. In Eastern Africa, the disease is considered endemic. Because of the geographic proximity and existing trade between eastern African countries and the Comoros archipelago, the latter is at risk of introduction and spread, and the first PPR outbreaks occurred in the Union of the Comoros in 2012. The objective of this study was to map the areas suitable for PPR occurrence and spread in the Union of the Comoros and four eastern African countries, namely Ethiopia, Uganda, Kenya, and Tanzania. A Geographic Information System (GIS)-based Multicriteria Evaluation (MCE) was developed. Risk factors for PPR occurrence and spread, and their relative importance, were identified using literature review and expert-based knowledge. Corresponding geographic data were collected, standardized, and combined based on a weighted linear combination to obtain PPR suitability maps. The accuracy of the maps was assessed using outbreak data from the EMPRES database and a ROC curve analysis. Our model showed an excellent ability to distinguish between absence and presence of outbreaks in Eastern Africa (AUC = 0.907; 95% CI [0.820–0.994]), and a very good performance in the Union of the Comoros (AUC = 0.889, 95% CI: [0.694–1]). These results highlight the efficiency of the GIS-MCE method, which can be applied at different geographic scales: continental, national and local. The resulting maps provide decision support tools for implementation of disease surveillance and control measures, thus contributing to the PPR eradication goal of OIE and FAO by 2030.

## Introduction

Peste des petits ruminants (PPR) is a highly contagious viral animal disease, mainly affecting domestic ruminants such as sheep and goats but also cattle and camels (1, 2). Captive or free wild ruminants can also be infected, including representatives of the Caprinae (wild goats, ibex, blue sheep), Antilopinae (gazelles, springbuck, saiga), Bovinae (buffalos, bushbuck, nilgai), Reduncinae (kobs, waterbucks), Hippotraginae (Oryx), Cephalophinae (duikers), Alcelaphinae (hartebeests), and Aepycerotinae (impalas) subfamilies (2–6). PPR is caused by a non-segmented negative strand RNA virus belonging to the *Morbillivirus* genus, family *Paramyxoviridae*, and as such closely related to rinderpest virus. The clinical phase is characterized by high fever, ocular and nasal discharge, pneumonia, dyspnea, and severe diarrhea (1), with mortality and morbidity rates as high as 90 and 100%. However, depending on the susceptibility of the population, as well as the virulence of the pathogen itself, severity of clinical signs may be highly variable (7).

PPR virus (PPRV) is transmitted by direct contact with infected animals through excretions (oral, nasal, feces) (8). The virus cannot survive for long outside the host. The infectious period is short, and animals either die or recover with a lifelong immunity (9). Within herds, PPRV disseminates between animals in close contact. Between herds, disease transmission occurs when sharing pastures and/or water points, and at live animal markets. Infected livestock movements are responsible for virus spread over medium or large distances (10). PPRV is also suspected to circulate silently, occasionally causing sporadic epidemics when the host population's immunity levels are low (2). In areas of Africa where the disease is endemic, PPR exhibits a seasonal pattern with an increased number of outbreaks at the beginning of the cooler wet season (11). This seasonality may be related to an increased survival and spread of the virus facilitated during the coldest months. Furthermore, as the dry season is a period of nutritional stress for ruminants due to the strong reduction in the quality and the availability of forage resources, physiological conditions and health status of animals are altered and their resistance to infections thus reduced by the end of the dry season/beginning of the wet season (12). Wildlife is often considered to play a negligible epidemiological role in PPRV persistence and spread (4, 9, 13). However, recent outbreaks of PPR in wild sheep and goat species indicate that PPRV is likely to be transmitted between domestic small ruminants and wild ungulates that share the same pasture. The potential and the direction of these spill-overs are still poorly understood (14).

**Figure 1:**
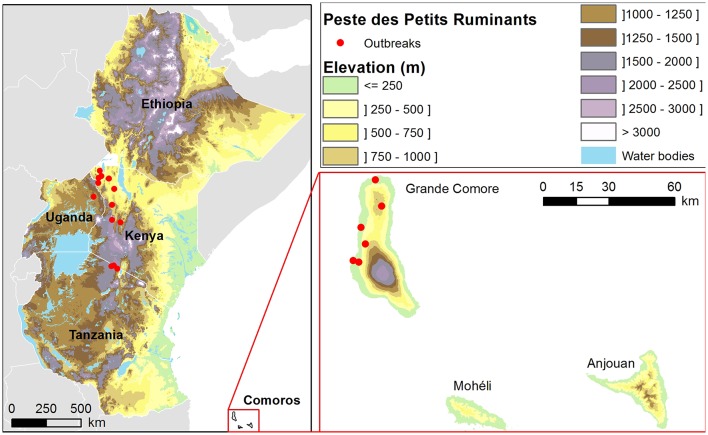
Location of the study area and peste des petits ruminants outbreaks (Source: EMPRES-i database) reported between 2007 and 2018 in Ethiopia, Uganda, Kenya, Tanzania and the Union of the Comoros.

PPRV was first described in West Africa in 1940 (15), and later recognized as endemic in West and Central Africa (16). The disease subsequently spread into Eastern Africa. The presence of PPR in Ethiopia has been clinically suspected since 1977 but was confirmed for the first time in 1991 from an outbreak near Addis Ababa (17) and is now endemic (18). The circulation of the disease across Eastern Africa was subsequently shown through the detection of the virus and/or of antibodies to PPRV in Kenya (1999), Uganda (2005 and 2007) and more recently in Tanzania (2010) (19–22). PPR is nowadays widely spread in the whole of Africa and is endemic in most eastern African countries (2, 23). In the Comoros archipelago, located in the Northern Mozambique Channel about 300 km of the southern coast of Tanzania (Figure 1), the first PPR outbreak occurred in 2012 (24). According to the results of phylogenetic analyses, this outbreak could be due to the introduction of goats infected by PPRV lineage III from the African continent (24). Although the outbreak was rapidly under control, the risk of re-emergence of PPRV remains high since the Union of the Comoros has strong livestock trading relationships with the African continent, importing large and small ruminants all year round (25).

In the Union of the Comoros islands and in Eastern Africa, livestock production is one of the main sources of income for the rural population. PPR is thus an important concern for poverty alleviation. Indeed, PPR has a huge economic impact, because of its high mortality rate for small ruminants, and high production losses caused by weight loss and abortions when animals are infected (26). The significant impacts on household-level livelihood, well-being and food security, as well as on rural communities and national economies have made PPR a priority for eradication (9). Due to the effective live attenuated vaccine producing lifelong immunity against the four PPRV lineages after a single administration (27), mass vaccination is recognized as one of the most important pillars of the future eradication of PPR. However, these mass vaccination campaigns are costly and difficult to implement due to several factors including lack of information and awareness regarding the disease, a sparse knowledge of the small ruminant population demography, a quick turnover of small ruminant population, and high mobility (9). Moreover, small ruminant population sizes may be huge in affected countries. Thus, the vaccination effort needs to be intense to maintain the population immunity high enough to eradicate the disease. Lastly, sheep and goats have a lower *per capita* value than cattle, and owners may be sometimes reluctant to invest money for vaccination for these animals. As stated in Mariner et al. (9), reaching an efficient vaccination coverage at a national level is hardly possible. Yet, targeted vaccination in endemic areas and in well-defined populations could be an efficient tool to eradicate the disease at the source. A recent modeling survey in Ethiopia suggested that viral spread could be prevented if the proportion of immune small ruminants is kept permanently above 37% in at least 71% of pastoral village populations (28). However, further spatiotemporal information in PPRV distribution identifying areas suitable for PPRV transmission and spread and on a larger scale is necessary.

Given the high risk of re-introduction of PPRV in the Union of the Comoros and its high level of endemicity in large territories of Eastern Africa, there is a need for tools which could help in prioritizing vaccination areas and optimizing allocation of limited resources. In this study we used expert knowledge and available geographic data to conduct a Geographic Information System (GIS)-based Multi Criteria Evaluation (MCE) to identify areas at risk of PPR occurrence and spread in the Union of the Comoros and in four countries of Eastern Africa: Tanzania because of its proximity to the Union of the Comoros, and Kenya, Uganda and Ethiopia because of their links with Tanzania through intensive intra-national and cross-border livestock trade. The objective was to provide a ready to use tool to implement PPR control strategies, in a context of PPR eradication by 2030 (29).

## Materials and Methods

### Spatial MCE Approach

GIS-based MCE is a process that transforms and combines geographical data and value judgments to obtain appropriate and useful information for decision making (30). The general approach of spatial MCE method and its applications in epidemiology have been detailed elsewhere (31–36). GIS-based MCE is particularly relevant in the absence of available or reliable field-based disease surveillance data, as it can be used to create preliminary maps that, while imperfect, may be used for risk-based surveillance (33).

The key stages of this method include (i) the identification of the factors, or criteria, that play a role in the risk to be mapped (e.g., risk of introduction, amplification, spread, maintenance, etc.), (ii) the weighting of these factors based on expert opinions or bibliographic knowledge, (iii) the collection of geographical data corresponding to the factors identified, and the creation of spatial, standardized suitability indices, (iv) the combination of the spatial suitability indices to produce a risk map.

### Identification of Environmental and Socio-Economic Factors for PPR Spread

Searches were performed in two journal databases (PubMed/Medline and ISI Web of Knowledge) with the keyword “peste des petits ruminants.” Papers were screened and articles dealing with the identification of risk factors were taken into account (Supplementary Table 1). From this bibliographic review, the following factors were identified as potentially associated with the transmission and spread of PPR in livestock in the Union of the Comoros and Eastern Africa:

- The density of small ruminants (goats, sheep): since PPRV is transmitted through direct contact between infected and susceptible animals, its spread is affected by host density;
- The proximity to water bodies: water bodies such as rivers can be a gathering point for livestock, and thus increase the risk of contact between animals of different herds;
- The movement of animals for trade and/or transhumance: animal movements are a major cause for long-distance spread of PPR;
- The density of roads: this factor is used as a proxy for short-distance animal movements for trade purpose;

Additional risk factors were identified for Eastern Africa only:

- The density of camels;
- The density of railways: this factor is used as a proxy for animal movements for trade purpose. Although railway is not the primary mode of livestock transportation—a lot of movements are on foot rather than by rail or road, it is used here as a proxy of the main axes linking cities which can be used by breeders and their animals to reach important livestock markets (37);
- The proximity to dry areas. Increasing risk is expected in dry and semi-dry areas where nomadic pastoralism is mostly practiced and more prone to livestock theft. Moreover, pastoralists often have larger herds than sedentary people -who can also rely on agriculture, and are less accessible to veterinarians;
- The proximity to protected areas as a proxy of the main areas where wild ungulates, a potential reservoir of PPRV, are present in large concentrations and where the wild/domestic ungulate interface occurs.

### Weighting of the Factors Associated With PPR Occurrence and Spread

The results of questionnaires addressed to 14 PPR experts (authors having published more than two scientific publications on PPR epidemiology and/or risk factors) (38) were used to generate the weights of the risk factors of PPR occurrence and spread in Africa through an Analytical Hierarchy Process (AHP) (39). With this method, experts compare two criteria at a time: (1) experts firstly specify whether risk factor A is more or less important than risk factor B and (2) they specify the degree of importance of factor A regarding factor B on a nine-point scale (factor A can be extremely more important, very strongly more important, strongly more important, moderately more important, equally important, moderately less important, strongly less important, very strongly less important or extremely less important than factor B), resulting in a pair-wise comparison matrix. A numerical weight is derived for each risk factor from the pair-wise comparison matrix, and a consistency ratio (CR) is determined (40–42). Details on the calculation of the CR are provided in Supplementary File 1. The final weight value of each risk factor is the median value of the *n* weight values determined by the *n* experts.

For the Union of the Comoros, the weights of the following risk factors were set to zero: camel density, railways density, proximity to dry areas, proximity to protected areas, as these risk factors are not relevant for this country. The weights of the other risk factors were proportionally increased such that the sum of the weights equaled 1.

### Collection of Geographical Data and Creation of Spatial, Standardized Suitability Indices

Geographical data were collected for each country (Figure 1) from different sources (Table 1), imported into a Geographic Information System (GIS), and processed to produce standardized spatial suitability indices with values ranging from 0 (completely unsuitable for occurrence and spread) to 1 (completely suitable) (GIS software: ESRI ArcGIS™, Spatial Analyst). The standardized spatial suitability indices for all countries were: sheep density, goat density, animal mobility index, road density, and proximity to water bodies. The animal mobility index was derived from the proximity to markets for eastern African countries, and from results from a mobility study for the Union of the Comoros (43). Additional standardized spatial suitability indices for eastern African countries were: camel density, railways density, proximity to wildlife national parks and proximity to dry areas. The calculation methods of the standardized geographical layers are provided in Table 2. At the end of the process, standardized spatial suitability indices were raster layers with pixel dimensions of 300 × 300 m, a good compromise between computational limitations due to the size of the study area (2,874,667 km^2^) and the spatial resolutions of the different datasets. The correlation between the different suitability indices was assessed (Supplementary Table 2). The resulting maps for the standardized spatial suitability indices are presented in Supplementary Figures 1, 2.

**Table 1:** Factors associated with the transmission of peste des petits ruminants in livestock populations for which spatial data were available, the hypothesized relationship between each factor and risk of transmission of PPR, and the source of geographic data.

**Criteria**	**Hypothesis**	**Data source**
Sheep and goat densities	Increasing small ruminant density is expected to be associated with a higher contact rate between susceptible and infected small ruminants and therefore greater risk of PPR spread	Geographic data: GADM database of Global Administrative Areas (http://www.gadm.org) Livestock data: national reports (Ethiopian central statistical agency 2013; Ministry of Agriculture United Republic of Tanzania 2012; Uganda Bureau of Statistics 2002; Ministry of Agriculture Kenya—Animal production division 2009; Comoros national census 2004)
Water bodies	Decreasing distance from water bodies is expected to be associated with increasing risk of spread of disease through increase contact among animals	Eastern Africa: FAO Africover—Rivers and wetlands (http://www.fao.org/geonetwork/) Comoros: data from EU project Global Climate Change Alliance “AMCC-Comores” (https://amcc-comores.info/)
Small ruminants' markets	Increasing density of animal movements or trading areas providing live or freshly slaughtered small ruminants is expected to be associated with increasing risk of spread of PPR	Uganda Bureau of statistics Kenya and Ethiopia: FAO data (http://kids.fao.org/glipha/)
Cities as proxy of small ruminants' markets		Tanzania: AFRIPOP data (https://www.worldpop.org)
Animal mobility		Comoros: 2012–2013 mobility data (43) and 2014–2015 mobility data (data collected in the framework of ANIMALRISK project)
Roads and railways	Increasing density of roads and railways is expected to be associated with increasing movements of small ruminants for trade, and thus a higher risk of spread of disease although there is no published evidence for the direct role of roads or railways in the spread of PPR.	Eastern Africa: Digital Chart of the World (http://divagis.org) Comoros: data from EU project Global Climate Change Alliance “AMCC-Comores” (https://amcc-comores.info/)
Camel density	Increasing density of camels may be associated with a greater risk of spread	Map of predicted camels distribution in Africa and Middle East countries 2006 (Source: FAO)
Dry and semi-dry areas, as proxy of pastoralism	Increasing risk would be expected in dry and semi-dry areas where nomadic pastoralism is mostly practiced	Global Land Cover Map: Globcover 2009 (http://due.esrin.esa.int/page_globcover.php)
Wildlife national parks, as proxy for wild ruminants densities	Proximity to wildlife national parks may be associated with increased risk of spread of PPR	World database on protected areas (https://www.protectedplanet.net/)

**Table 2:** Details of the geographic information systems manipulations required to convert the collected data into risk factor layers.

**Suitability index raster**	**Collected data associated to PPRV transmission suitability**	**GIS manipulation**	**Scaling function**
Sheep density	Districts (polygons) Table with number of sheep per district	Join geographic layer and table Calculate animal densities (nb animal/km^2^)	Positive linear relationship
Goat density	Districts (polygons) Table with number of goats per district	Join geographic layer and table Calculate animal densities (nb animal/km^2^)	Positive linear relationship
Animal mobility	Small ruminants' markets of Uganda, Ethiopia, and Kenya (points)	Calculate and map distance (km) to markets	Sigmoidal, monotonically decreasing relationship between 0 and 50 km, with negligible risk after 50 km
	Tanzania: population map (spatial resolution 0.000833333°~900 m at the latitude of the study area)	Calculate and map distance (km) to areas with population densities >1000 inhab./km^2^, with elevation map^a^ as cost map	Sigmoidal, monotonically decreasing relationship between 0 and 50 km, with negligible risk after 50 km.
	Comoros: Districts (polygons) Table with number of imported animals per district	Join geographic layer and table	Positive linear relationship
Proximity to water bodies	Rivers and wetlands (polylines and polygons)	Calculate and map distance (km) to rivers and wetlands, with elevation map as cost map	Sigmoidal, monotonically decreasing relationship between 0 and 50 km, with negligible risk after 50 km.
Road density	Roads (polylines)	Calculate and map density of roads per 100 km^2^	Positive linear relationship
Railways density	Railways (polylines)	Calculate and map density of railways per 100 km^2^	Positive linear relationship
Camel density	Camel density map (resolution 0.000833333° ~900 m)	No manipulation required	Positive linear relationship
Proximity to dry areas	Land cover map (resolution 300 × 300 m)	Extract dry areas, calculate and map distance (km) to dry areas, with elevation map as cost map	Sigmoidal, monotonically decreasing relationship between 0 and 50 km, with negligible risk after 50 km
Proximity to wildlife national parks	Wildlife national parks (polygons)	Calculate and map distance (km) to: Conservation Area, Controlled Hunting Area, Game Controlled Area, Game Reserve, Game sanctuary, Hunting reserve, National Park, National Reserve, Nature Reserve, Sanctuary, Wildlife Reserve. Use elevation map as cost map	Sigmoidal, monotonically decreasing relationship between 0 and 100 km, with negligible risk after 100 km.

a *Source of elevation data: Shuttle Radar Topographic Mission (SRTM) downloaded from http://srtm.csi.cgiar.org/srtmdata/*.

### Combination of the Spatial Suitability Indices

The standardized spatial suitability indices for PPR occurrence and spread (sheep density, goat density, animal mobility index, road density, proximity to water bodies, camel density, railways density, proximity to wildlife national parks, and proximity to dry areas) were combined using a weighted linear combination (WLC) with their corresponding weights (41). The resulting map is a suitability map for PPR occurrence and spread, with pixel values ranging from 0 (completely unsuitable) to 1 (completely suitable).

### Assessment of the PPR Suitability Map

PPR outbreaks reported and geo-located between 2007 and 2018 were used to assess the consistency of PPR risk maps (Source: EMPRES-i database). In total, 23 PPR outbreaks, so called “presence,” were recorded, including 13 outbreaks in Kenya, 3 in United Republic of Tanzania, 1 in Uganda, and 6 in Union of the Comoros (Figure 1, Supplementary Table 3). As no outbreaks were reported in Ethiopia, the assessment of the PPR suitability map could not be performed for this country.

One hundred locations of disease “pseudo absence” were randomly generated in the countries where PPR outbreaks occurred in continental Africa (Uganda, Kenya, and Tanzania), under the condition of being 25 km distant from other “absence” or “presence” locations (Supplementary Figure 3). In the Union of the Comoros, 6 locations of PPR “pseudo absence” were randomly generated in the Grande Comore Island, under the condition of being 5 km distant from other “absence” or “presence” locations, taking into account the size of the Comoros islands (Figure 1).

Then, the value of the quantitative suitability estimates for PPR occurrence and spread was extracted for each “presence” or “pseudo absence” location. The AUC (area under curve) of the ROC curve (44) was calculated to evaluate the capacity of the model to distinguish “presence” from “absence” locations with good predictive accuracy. The suitability maps were evaluated separately for (i) continental countries where outbreaks were reported (i.e., Kenya, Tanzania and Uganda) and (ii) the Union of the Comoros (pROC package, R software, https://cran.r-project.org/web/packages/pROC/index.html).

## Results

The resulting weights of the factors associated with PPR occurrence and spread in the four countries of Eastern Africa and in the Union of the Comoros are presented in Table 3.

**Table 3:** Weights of the factors associated with risk of PPR outbreaks in Eastern Africa and the Union of the Comoros [in brackets: minimum and maximum weight values obtained from the questionnaires].

	**Ethiopia, Kenya, Tanzania, Uganda**	**Union of the Comoros**
Goat density	0.255 [0.180–0.345]	0.357 [0.224–0.490]
Sheep density	0.225 [0.135–0.276]	0.315 [0.192–0.387]
Road density	0.100 [0.020–0.301]	0.140 [0.062–0.456]
Proximity to water bodies	0.069 [0.015–0.077]	0.096 [0.028–0.172]
Animal mobility index	0.066 [0.044–0.161]	0.092 [0.058–0.093]
Proximity to dry areas	0.108 [0.030–0.127]	0
Camel density	0.094 [0.043–0.164]	0
Proximity to wildlife national parks	0.042 [0.021–0.171]	0
Railways density	0.041 [0.015–0.081]	0

According to results of questionnaires, small ruminant densities were identified as the most important factors for PPR circulation and spread for all countries. In Eastern Africa, the proximity to dry areas was identified as the next important factor, followed by (in decreasing order) road density, camel density, proximity to water bodies, animal mobility index, proximity to wildlife parks, and railways density. In the Union of the Comoros, the animal mobility index and the road density were identified as important factors, followed by the proximity to water bodies.

**Figure 2:**
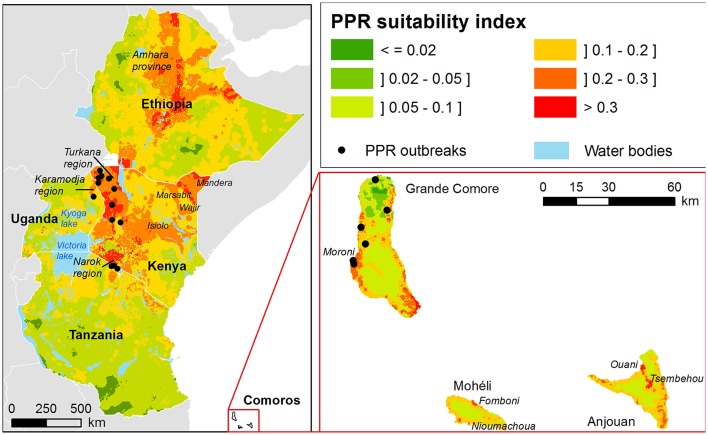
Suitability maps for PPR outbreaks in Eastern Africa and the Union of the Comoros.

Figure 2 presents the suitability map of PPR occurrence in Ethiopia, Kenya, Tanzania, Uganda, and the Union of the Comoros produced from the MCE process. In the text, we refer to green areas on the map (Figure 2) as very low (below 0.05) and low (between 0.05 and 0.1) risk for PPR occurrence and spread, and to yellow, orange, and red areas as medium, high and very high risk, respectively.

According to our model, in Ethiopia areas at high risk of occurrence and spread are mainly located in the highlands. The rest of the country is at medium risk, except for the horn of Ethiopia, and lowlands located in the south as well as in areas neighboring Sudan.

In Kenya, almost the whole country appears to be at risk, except the fertile plateau of the southeastern part. Areas identified at high risk by the model are mainly located on the northwestern part, including the Turkana region, an area constituting a mixed landscape with high altitudes plateau and lowland in the extreme north. A second area at risk is identified in a lowland region at the northeast side of the country. A third pocket at risk is highlighted in the center of the country, this region including both highlands and lowlands. The last area at risk, the Narok region, is neighboring Tanzania.

In Uganda, few areas are identified at high risk by the model, except a small region in the north east, overlapping with Karamodja region. North of Lake Victoria and areas surrounding Lake Kyoga are identified at medium risk, with some additional pockets in the southern extremity of the country, near Rwanda.

The majority of Tanzania is identified as medium or low risk by the model, except in the northeastern part of the country neighboring the boundary with Kenya.

In contrast to Eastern Africa, in the Union of the Comoros high risk areas are located mostly along the coast at low elevations. In Moheli, only very restricted areas are at high risk: the high risk areas correspond to the regions around the two largest cities of the island, Fomboni and Nioumachoua. According to the model, the rest of the island is at middle to low risk for PPR occurrence and spread. In Anjouan the main area at risk for PPR suitability ranges from the North coast around the city of Ouani to the third largest city of the island, Tsembehou, located in a natural circus, a circular steep-sided hollow, in the middle of the island. The risk of PPR occurrence is more variable in Grande Comore. The main high risk areas are located around Moroni, the capital city, and in the southern extremity of the island. The northern part of the island is at low to very low risk for PPR transmission and spread, except the East coast.

**Figure 3:**
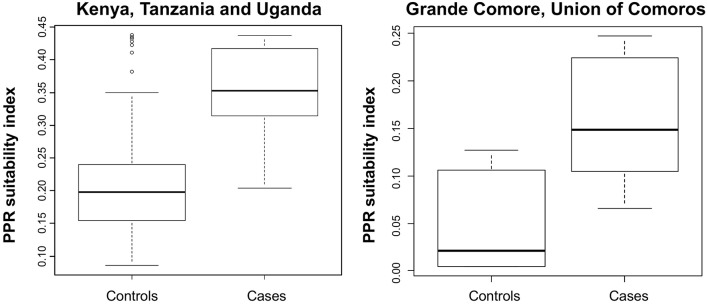
Assessment of the suitability index for PPR occurrence in livestock in continental Eastern Africa countries where PPR outbreaks occurred (Kenya, Uganda, Tanzania) and in the Union of the Comoros. Box-plots showing PPR occurrence suitability index values for cases (PPR outbreak locations) and controls (random “pseudoabsence” locations) in continental Africa (left panel) and in the Union of the Comoros (right panel). Box-plots show median values (solid horizontal line), 50th percentile values (box-plot outline), 90th percentile values (whiskers), and outlier values (open circles).

The ROC AUC associated with the suitability map for PPR occurrence and spread in Eastern Africa (Kenya, Tanzania and Uganda) demonstrated the capacity of the model to distinguish “presence” from “absence” locations with very good predictive accuracy (AUC = 0.891; 95% CI [0.821–0.960]) (Figure 3).

According to the ROC analysis, the suitability map for PPR spread in Grande Comore, Union of the Comoros showed a very good fitting (AUC = 0.889, 95% CI: [0.694–1]).

## Discussion

The identification of areas at-risk for PPR is essential for implementing risk-based surveillance and control measures. To our knowledge, this is the first study aiming to produce regional suitability maps for PPR using GIS-MCE method combined with outbreak dataset validation.

As a whole, there is a good consistency between areas identified at risk by the model and what is known about PPR circulation in the five countries of interest. In the next paragraphs, we discuss in detail the comparison of the obtained suitability maps and results of previous epidemiological studies, for each studied country. Regarding the eastern African countries, one should keep in mind that the number of outbreaks used for validation is very low compared to the surface of the territories studied. In addition, PPR outbreaks may be underreported in the four eastern African countries, where the disease is endemic and thus declaration non-mandatory. The validation results should therefore be taken with caution, and regularly re-assessed, as more cases are reported.

In Kenya, the Turkana region located in the north-western part of the country is identified as high risk: in 2011 in this area, PPRV Lineage III was detected from tissue samples collected from goats suspected of having died of PPR (45). The results of a seroprevalence study showed that 40% of the sheep (*n* = 431) and 32% of the goats (*n* = 538) sampled were seropositive (46). In 2016, PPR occurrence was confirmed in both camels and goats in the second main area identified at risk by our model, i.e., the north-eastern part of the country including Mandera, Wajir, Isiolo, and Marsabit districts (22).

In Tanzania, seropositive cases were found in the north-eastern part of the country during a nationwide surveillance in 2008–2013: this region is identified as a high to medium risky area by the model. As emphasized by Spiegel and Havas (47), the Tanzanian outbreak overlapped with trade routes that travel through Nakuru, in south-west Kenya, and go all the way to Nairobi and down into Tanzania. However, some cases were detected in the south of the Lake Victoria, identified as low risk by our model (23).

Little is known about PPR circulation in Uganda. Nevertheless, in 2007–2008 an outbreak occurred in sheep and goats in Karamodja region identified as at risk by our model. A survey was performed to characterize this outbreak: of the 338 small ruminants sampled, 38.1% (26/67) and 13.0% (41/316) of samples were found positive by PCR in 2007 and 2008, respectively (48).

PPR is known to be endemic in Ethiopia. However, few studies have been undertaken to document areas where the virus is currently circulating. The largest serosurvey, performed in 1999, demonstrated high seroprevalence rates in the Afar, Amhara, Oromia, Southern Nations, Nationalities, and Peoples' Region (SNNPR), Somali Tigray and Benishangul Gumuz regions (11). According to this work, areas of low altitudes, where pastoralists prevail, were more affected than highlands that are home to sedentary mixed livestock–crop farms (11). In 2010, lineage IV PPRV was isolated in Amhara region and a further study performed in eastern Amhara provides evidence of the continued spread of the same lineage in this area (49, 50). Our model, in accordance with these findings, identified the eastern Amhara as at high risk of PPR occurrence (Figure 2). Moreover, high seroprevalence of PPRV in small ruminants was reported in neighboring lowland pastoral areas (11, 18, 51, 52), identified at medium risk by our model. According to our model, highlands are at higher risk for PPR outbreaks than adjacent areas (Figure 2), which seems in apparent contradiction with the cited serosurveys. Yet, it should be noted that a large part of highlands could not be included in the sampling frame of the national serosurvey (11): high risk areas may have been missed. On the other hand, our results are consistent with a recent modeling study suggesting that the pastoral production system, mainly located in the lowlands act as a PPRV reservoir, and that the virus frequently spreads to the highlands through herd movements (28). Finally, it should be noted that (i) updated livestock census data would certainly improve the predictive accuracy of the model and (ii) the construction of GIS-MCE maps at regional scale may introduce a bias in suitability predictions at national level (with weights discussed with national experts). In particular, the weight of pastoral areas may be underestimated, which will strongly impact the suitability maps in Ethiopia, where pastoralism covers large areas.

In the Union of the Comoros, our model shows a good performance, although only few data from the 2012 outbreak were available. The 2012 outbreak was rapidly controlled, thanks to farmer practices, who slaughtered animals as soon as the first clinical signs were described by the animal health local authorities (24). These control measures limited the spread of PPR, and no outbreaks were reported in some areas identified at risk by our model (Figure 2). The majority of the Union of the Comoros is predicted to be at low risk by our model, which is in agreement with what one would expect. Indeed, farmers are sedentary and usually own few animals; the small ruminant population of the three islands is around 110,000 according to the 2004 census. The volcanic topography and the paucity of water bodies are likely to limit direct contact among animals. These factors play in favor of a lower risk for PPR spread in Union of the Comoros. However, because of the proximity with Eastern Africa and the historical trade link between the two areas, the Union of the Comoros remains at risk of introduction. Around 3,000 small ruminants are introduced every year in Moroni (Figure 2), capital city of Comoros and main entry port for livestock from continental Africa. Most of the time, these animals are bought and slaughtered for “Grand Mariages” ceremonies, part of the Comorian culture. In some cases, animals are kept for improving the genetic level of herds. There is no quarantine on arrival and the animals are first kept in small size facilities belonging to the importers for few days before being moved either to local slaughtering places or introduced into a herd (53). In this context, combining the identification of areas at risk of PPR transmission with an assessment of the risk of introduction in the one main entry port, would allow the targeting of surveillance measures and a better allocation of available funds to limit pathogens introduction due to animal importations.

Studies have shown that PPR outbreaks are related to factors that promote hosts contacts such as livestock trade, husbandry practices, nomadism, as well as socio-economic and ecological factors (47, 54). In this work, we aimed to integrate all known risk factors and available associated geographic data. The main limitations of the produced maps are firstly related to the quality of the data used as risk factors. Small ruminant density is the most important risk factor in our study. In resource limited settings, animal censuses are often partial, occurring too rarely, leading to poor and not up-to-date data. More precise census data accounting, for example, for the number of animals per holding and its geographical location, would increase the precision of the resulting risk map. Secondly, as animal movements play a crucial role in the long distance spread of livestock disease (55), instead of using proxies (i.e., the proximity to markets), an exhaustive knowledge of the movement networks would improve the spatial risk factor for animal mobility. Third, the occurrence of outbreaks is strongly linked in endemic areas to interventions and control measures in place, as well as the immunity of the population: survival after infection resulting in lifelong immunity, herd population dynamics and renewal rates, as well as individual and herd level immunity are key factors that largely modulate the circulation of the virus in a given area. These factors were not incorporated in our model and this can result in discrepancies between the predicted suitability maps and results of epidemiological studies. Fourth, it must be stressed that our results include uncertainties inherent in the expert-based approach, regarding the relative importance of the different risk factors (Table 3). In the future, the weights of the different factors have to be re-evaluated according to scientific knowledge development (Supplementary Table 1), in particular regarding the role of camels or wildlife in PPR transmission, for which there is so far little evidence. Finally, it is worth noting that we used random generated “disease absence locations” for computing ROC AUC, assuming that absence of reported outbreaks is likely to result from an absence of the pathogen, which may not be true. The use of more extensive serological data from future studies, national surveillance datasets, or information extracted from published reports (56) could be easily integrated in the framework of the model validation and would make our results stronger.

In our study, the GIS-MCE method was applied to territories with different sizes, including small islands and continental countries. Our results demonstrated the effectiveness of the GIS-MCE method, which has been successfully applied at different geographic scales and settings. Indeed, the validation of the suitability maps using reported PPR outbreaks produced good results according to the ROC AUC method. These results suggest that the output regional-scale suitability maps have a reasonable predictive accuracy and could be used for risk-based surveillance and control purposes. As shown by rinderpest eradication, regional and international approaches should be combined for transboundary animal diseases (57, 58). The GIS-MCE method developed in this study intended to respond to this need. Applied to PPR at regional and international scales, it enables discussion among stakeholders and experts from the different countries concerned, and a comprehensive overview of the disease suitability areas.

In conclusion, the knowledge-driven approach proposed in this work to map the areas suitable for PPR occurrence and spread in Eastern Africa and Union of the Comoros provide valuable tools for several purposes: (i) to integrate the available knowledge about the disease; (ii) to provide suitability maps at regional scale using free geographic data. Applied in different geographical and epidemiological contexts (33, 34, 36, 59, 60), such an approach allows straightforward and easy updating of maps for users by including more precise geographic data, newly described risk factors, by modifying the weights of each factor, or by comparing scenarios of transmission. Another perspective of this work deals with the combination of the produced PPR suitability maps with modeling approaches accounting for temporal variability and transmission processes (28), in order to provide recommendations for vaccination strategies.

## Data Availability Statement

The datasets and PPR suitability maps of this study are available on request to the corresponding author.

## Author Contributions

AT, VC, EC, and CC-S: conceptualization. AT, A-SR, and AW-S: data curation. AT, VC, A-SR, and AW-S: formal analysis. AT, VC, and AW-S: methodology. VC, EC, and CC-S: project administration. AT, VC, A-SR, AW-S, YM, and OC: resources. AT and A-SR: software and visualization. VC and CC-S: supervision. AT, VC, and A-SR: validation and writing—original draft preparation. All co-authors: writing—review and editing.

### Conflict of Interest

The authors declare that the research was conducted in the absence of any commercial or financial relationships that could be construed as a potential conflict of interest.
