# Supplementary material for: Spatial Multicriteria Evaluation for Mapping the Risk of Occurrence of Peste des Petits Ruminants in Eastern Africa and the Union of the Comoros
Source: Front Vet Sci. 2019 Dec 12;6:455. doi: 10.3389/fvets.2019.00455 (PMC6922030; doi:10.3389/fvets.2019.00455)
Supplement: Supplementary file 1 [file Data_Sheet_1.PDF]

Spatial Multicriteria Evaluation for mapping the risk of outbreak of Peste des Petits Ruminants in Eastern Africa and the Union of the Comoros

Anne-Sophie Ruget, Annelise Tran, Agnès Waret-Szkuta, Youssouf Ousseni Moutroifi, Onzade Charafouddine, Eric Cardinale, Catherine Cêtre-Sossah, Véronique Chevalier

Supplementary Material

1 Table S1. Factors associated with the transmission of Peste des Petits Ruminants in small ruminants as identified by the published literature review

	Factor[*]	Reference
**Individual level**	Species	(Lefevre and Diallo, 1990; Roeder et al., 1994; FAO, 1999; Diallo, 2000; Awa et al., 2002; Ozkul et al., 2002; Singh et al., 2004; Abraham et al., 2005; Taylor and Ali, 2005; Sow et al., 2008; Abubakar et al., 2011)
	Breed	(Odo, 2003; Lundervold et al., 2004; Bazarghani et al., 2006)
	Age	(Tounkara et al., 1996; Sow et al., 2008; Abubakar et al., 2009)
	Sex	(Abubakar et al., 2009)
**Production systems**	**Pastoralism, nomadic pastoralism***	(Nanda et al., 1996; Shankar et al., 1998; Singh et al., 2004; Abubakar et al., 2009; Bett et al., 2009; Abubakar et al., 2011; Spiegel and Havas, 2019)
	**Large herds, high ruminant densities***	(Ozkul et al., 2002; Al-Majali et al., 2008; Khan et al., 2008)
	**Introduction of animals purchased at the market***	(Singh et al., 2004; Abubakar et al., 2009; Abubakar et al., 2011)

[*] Risk factors for which spatial data were available and included in the study are indicated by an asterisk.

	Mixed herds (sheep and goats)	(Anderson and McKay, 1994; Al-Majali et al., 2008)
	**Presence of wild ruminants***	(Furley et al., 1987; Anderson, 1995; Abu-Elzein et al., 2004; Couacy-Hymann et al., 2005; Banyard et al., 2010; Kinne et al., 2010; Lembo et al., 2013; Munir, 2014)
**Markets / trade / theft**	**Importation of live small ruminants***	(Al-Naeem et al., 2000; Ozkul et al., 2002; Al-Dubaib, 2009)
	Animal movements between neighboring countries	(Ozkul et al., 2002; Singh et al., 2004; Osman et al., 2009; Wang et al., 2009; Spiegel and Havas, 2019)
	**Visiting live animal markets***	(Martrenchar et al., 1995; Shankar et al., 1998; Al-Majali et al., 2008)
	Festival periods	(Bonniwell, 1980)
	Traditional and commercial practices	(Bazarghani et al., 2006; Spiegel and Havas, 2019)
	Livestock theft	(Bett et al., 2009)
**Climate**	Season	(Taylor, 1984; Diallo, 2003; Odo, 2003; Singh et al., 2004; Gopilo, 2005; Waret-Szkuta et al., 2008; Abubakar et al., 2009)
**Veterinary services**	Inefficient quarantine<br>Limited availability and access to veterinary services<br>Lack of surveillance systems	(Bazarghani et al., 2006; Al-Majali et al., 2008; Bett et al., 2009)

Table S2. Correlation matrix for Peste des Petits Ruminants suitability indices

Index*	Camel	Dry areas	Goats	Mobility	Parks	Railways	Roads	Sheep	Water
Camel	1	0.32	0.01	-0.07	0.02	-0.03	-0.03	-0.01	-0.13
Dry areas	0.32	1	0.20	-0.14	0.04	-0.17	-0.03	0.32	-0.37
Goats	0.01	0.20	1	0.18	0.14	-0.09	0.02	0.51	-0.04
Mobility	-0.07	-0.14	0.18	1	-0.02	0.23	0.33	0.16	0.09
Parks	0.02	0.04	0.14	-0.02	1	0.16	-0.06	0.07	0.04
Railways	-0.03	-0.17	-0.09	0.23	0.16	1	0.26	-0.13	0.14
Roads	-0.03	-0.03	0.02	0.33	-0.06	0.26	1	0.16	0.01
Sheep	-0.01	0.32	0.51	0.16	0.07	-0.13	0.16	1	-0.11
Water	-0.13	-0.37	-0.04	0.09	0.04	0.14	0.01	-0.11	1

* Sheep: sheep density; Goat: goat density; Camel: camel density; Mobility: Animal mobility index; Roads: density of roads; Railways: density of railways; Water: proximity to water bodies; Parks: proximity to wildlife national parks; Dry areas: proximity to dry areas

3 **Table S3. PPR outbreaks reported and geo-located between 2007 and 2018 (Source: EMPRES-i database).**

Country	Admin1	Locality name	Latitude	Longitude	Reporting date
Comoros	Ngazidja	Topping-Chezani	-11.7148	43.2664	14/01/2013
Comoros	Ngazidja	Dzahani	-11.6425	43.2938	14/01/2013
Comoros	Ngazidja	Mbni-SadaMouwamboi-Dimadjou	-11.4901	43.3599	14/01/2013
Comoros	Ngazidja	Ouella-Batsa	-11.3843	43.3348	14/01/2013
Comoros	Ngazidja	Hamanvou	-11.576	43.275	14/01/2013
Comoros	Ngazidja	Moroni	-11.7092	43.2433	14/01/2013
United Republic of Tanzania	Arusha	Ngorongoro	-1.9431	35.4285	20/05/2009
United Republic of Tanzania	Arusha	Soitasambu	-2.1044	35.8048	20/05/2009
United Republic of Tanzania	Arusha	Soitasambu	-1.9	35.583	30/03/2009
Uganda	Napak	unknown	2.591377	34.26382	30/07/2007
Kenya	Rift Valley	Baringo	0.921508	36.0156	06/01/2013
Kenya	Rift Valley	Marakwet	1.083492	35.4578	06/01/2013
Kenya	Rift Valley	Kaputir / Kakong	2.06	35.46	16/05/2012
Kenya	Rift Valley	Kaputir	2.0667	35.4667	16/05/2007
Kenya	Rift Valley	Meiyan	3.117	35.6	02/10/2008
Kenya	Rift Valley	Lokamaringyang	3.117	35.6	02/10/2008
Kenya	Rift Valley	Letea	3.779	35.256	27/03/2007
Kenya	Rift Valley	Nanam	4.3	34.6833	27/03/2007
Kenya	Rift Valley	Lokangae	3.779	35.256	27/03/2007
Kenya	Rift Valley	Moruarengan	3.779	35.256	27/03/2007
Kenya	Rift Valley	Loreng	3.5	34.5666	27/03/2007
Kenya	Rift Valley	Songot	3.9475	34.776	15/01/2007
Kenya	Rift Valley	Natiira	3.8241	34.5988	15/01/2007

4 **Figure S1.**

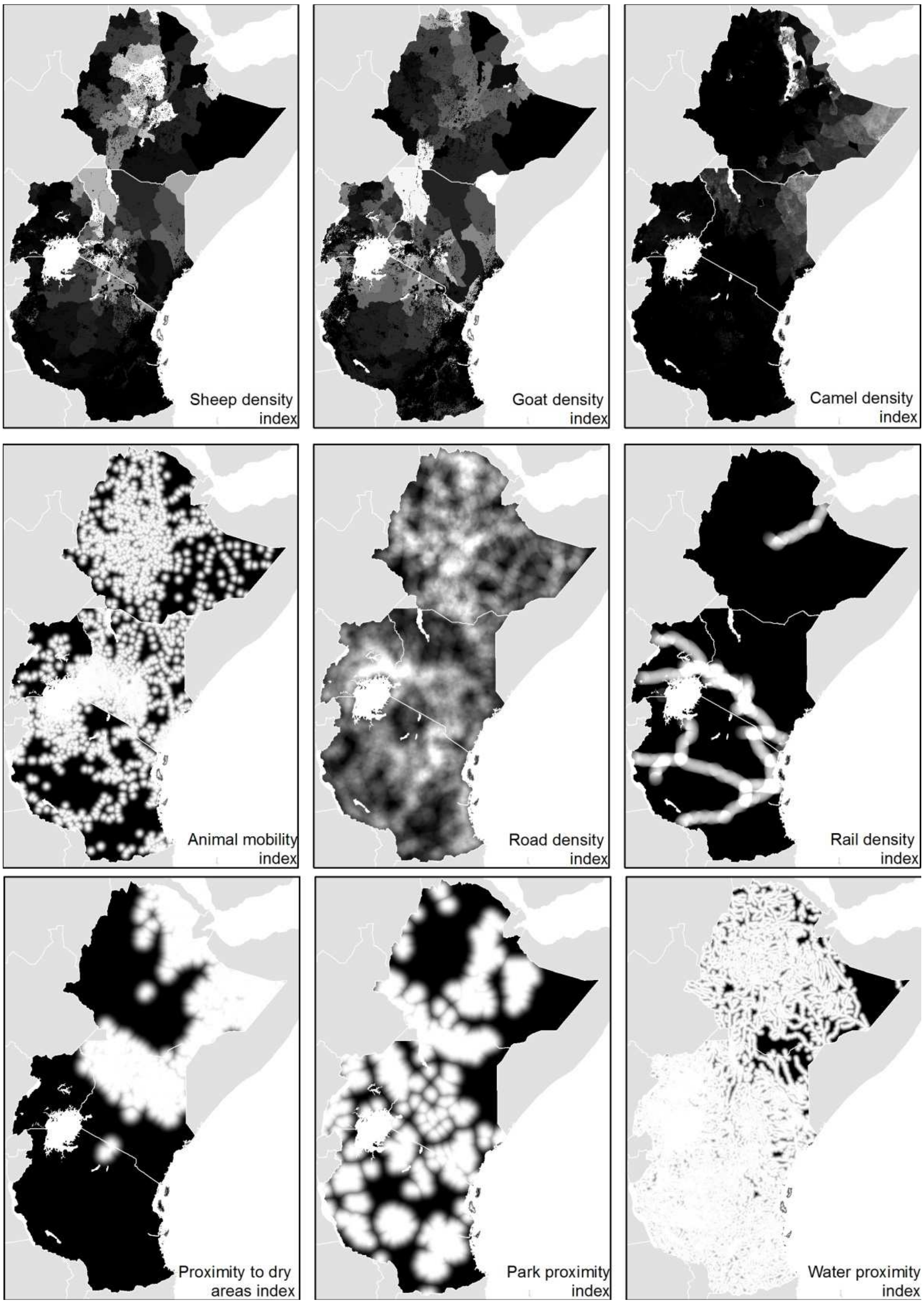

Supplementary Figure 1. Standardized spatial PPR suitability indices in Ethiopia, Kenya, Uganda and Tanzania.

5 Figure S2

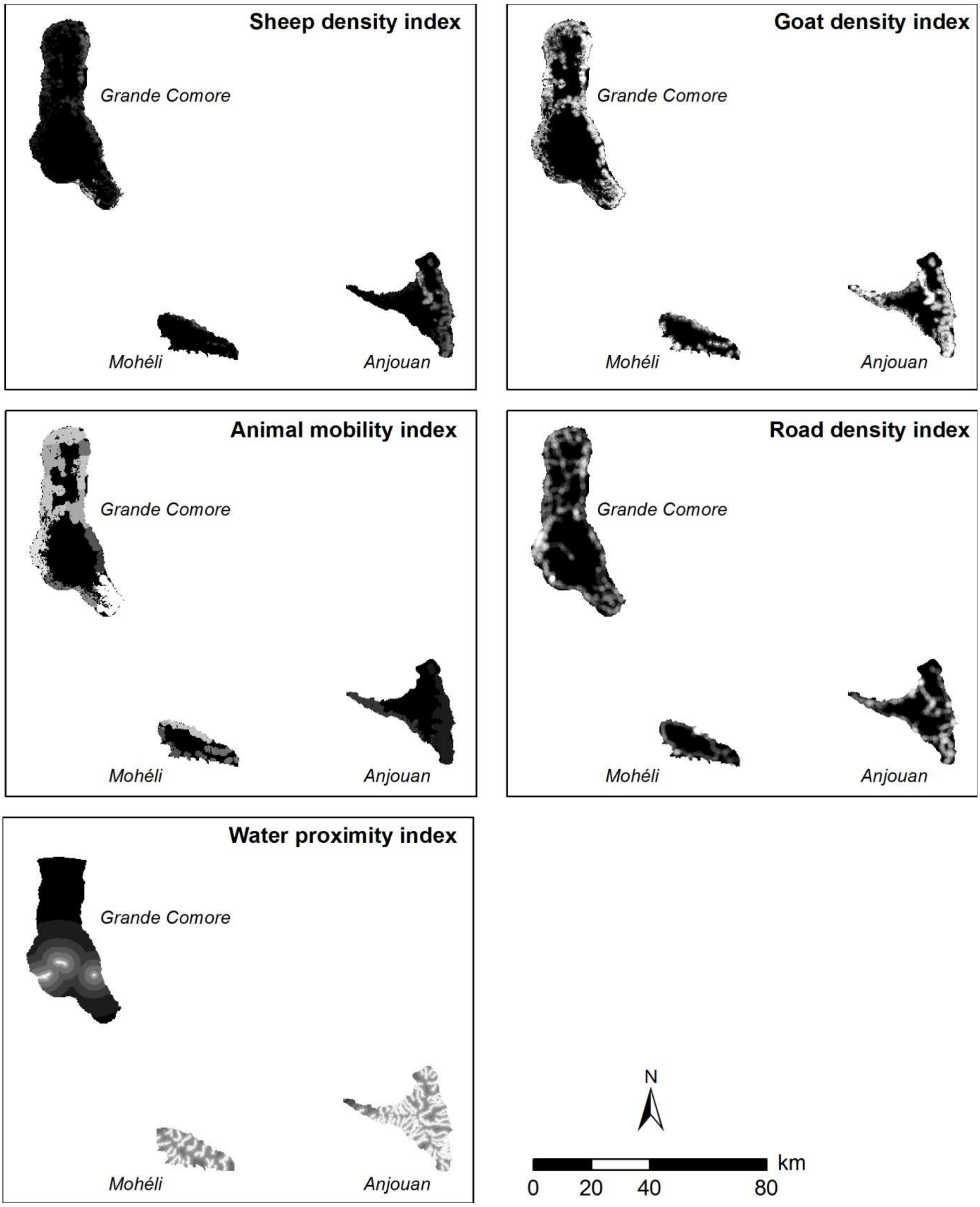

Supplementary Figure 2. Standardized spatial PPR suitability indices in Union of the Comoros.

6 Figure S3

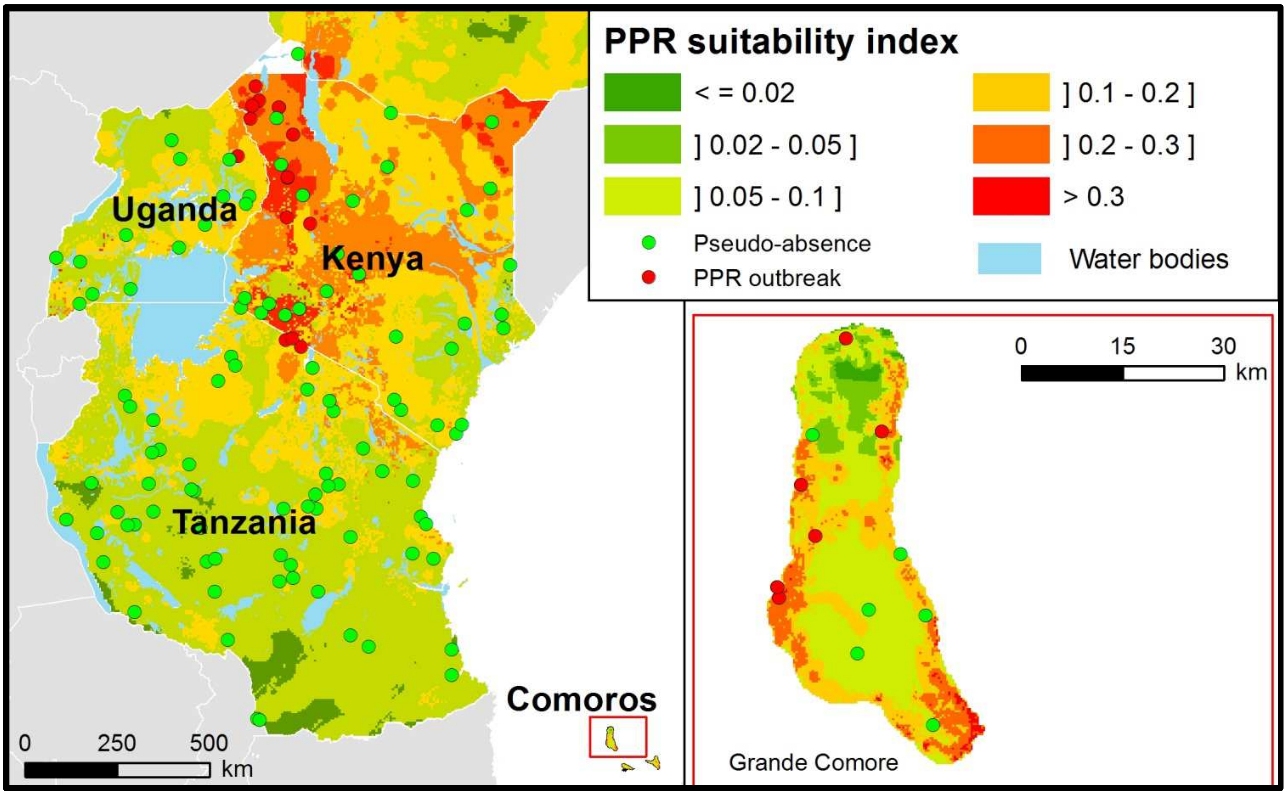

Supplementary Figure 3. Location of random pseudo-absence locations used for validation, Kenya, Uganda Tanzania, and Grande Comore.

7 File S1. Consistency ratio calculation

The consistency ratio (CR) aims at verifying the consistency of the pairwise comparison matrix and evaluating the probability that the weights were randomly generated. We calculated the CR following the method described by (Rao, 2013) and detailed in (Drobne and Lisec, 2009).

The CR is defined as follows:

$$CR = \frac{CI}{RI} \qquad (1)$$

where CI is the *Consistency Index* and RI the *Random consistency Index*.

CI is the degree of logical consistency among pairwise comparison. The smaller the value of CI, the smaller is the deviation from consistency.

$$CI = \frac{\lambda max - M}{M-1} \qquad (2)$$

where λmax is the maximum eigenvalue of the pairwise comparison matrix.

λmax is calculated by achieving the following operations:

- Sum the values in each column of the pairwise comparison matrix
- Compute the normalized pairwise comparison matrix by dividing each element of the pairwise comparison matrix by its column total
- Sum the rows of the normalized pairwise comparison matrix
- Multiply the weight of the first factor times its row total. λmax is the sum of these values

M is the size of the comparison matrix (in our case, M = 5 in the Union of the Comoros, and M=9 in Eastern Africa).

RI is the average CI value of randomly generated comparison matrices

The table below presents the RI values for different sizes of the comparison matrix (Saaty, 1977) (in bold, the values used on our study):

Random Consistency	Matrix Size (M)									
	1	2	3	4	**5**	6	7	8	**9**	10
RI	0.00	0.00	0.58	0.90	**1.12**	1.24	1.32	1.41	**1.45**	1.49

8 References

Abraham, G., Sintayehu, A., Libeau, G., Albina, E., Roger, F., Laekemariam, Y., et al. (2005). Antibody seroprevalences against peste des petits ruminants (PPR) virus in camels, cattle, goats and sheep in Ethiopia. *Prev Vet Med* 70(1-2)**,** 51-57. doi: 10.1016/j.prevetmed.2005.02.011.

Abu-Elzein, E.M., Housawi, F.M., Bashareek, Y., Gameel, A.A., Al-Afaleq, A.I., and Anderson, E. (2004). Severe PPR infection in gazelles kept under semi-free range conditions. *J Vet Med B Infect Dis Vet Public Health* 51(2)**,** 68-71.

Abubakar, M., Jamal, S.M., Arshed, M.J., Hussain, M., and Ali, Q. (2009). Peste des petits ruminants virus (PPRV) infection; its association with species, seasonal variations and geography. *Trop Anim Health Prod* 41(7)**,** 1197-1202. doi: 10.1007/s11250-008-9300-9.

Abubakar, M., Khan, H.A., Arshed, M.J., Hussain, M., and Ali, Q. (2011). Peste des petits ruminants (PPR): Disease appraisal with global and Pakistan perspective. *Small ruminant research* 96(1)**,** 1-10.

Al-Dubaib, M.A. (2009). Peste des petits ruminants morbillivirus infection in lambs and young goats at Qassim region, Saudi Arabia. *Trop Anim Health Prod* 41(2)**,** 217-220.

Al-Majali, A.M., Hussain, N.O., Amarin, N.M., and Majok, A.A. (2008). Seroprevalence of, and risk factors for, peste des petits ruminants in sheep and goats in Northern Jordan. *Prev Vet Med* 85(1-2)**,** 1-8.

Al-Naeem, A., Abu Elzein, E.M., and Al-Afaleq, A.I. (2000). Epizootiological aspects of peste des petits ruminants and rinderpest in sheep and goats in Saudi Arabia. *Rev Sci Tech* 19(3)**,** 855-858.

Anderson, E.C. (1995). Morbillivirus infections in wildlife (in relation to their population biology and disease control in domestic animals). *Vet Microbiol* 44(2-4)**,** 319-332.

Anderson, J., and McKay, J.A. (1994). The detection of antibodies against peste des petits ruminants virus in cattle, sheep and goats and the possible implications to rinderpest control programmes. *Epidemiol Infect* 112(1)**,** 225-231.

Awa, D.N., Ngagnou, A., Tefiang, E., Yaya, D., and Njoya, A. (2002). Post vaccination and colostral peste des petits ruminants antibody dynamics in research flocks of Kirdi goats and Foulbe sheep of north Cameroon. *Prev Vet Med* 55(4)**,** 265-271.

Banyard, A.C., Parida, S., Batten, C., Oura, C., Kwiatek, O., and Libeau, G. (2010). Global distribution of peste des petits ruminants virus and prospects for improved diagnosis and control. *J Gen Virol* 91(Pt 12)**,** 2885-2897. doi: 10.1099/vir.0.025841-0.

Bazarghani, T.T., Charkhkar, S., Doroudi, J., and Bani Hassan, E. (2006). A Review on Peste des Petits Ruminants (PPR) with Special Reference to PPR in Iran. *J Vet Med B Infect Dis Vet Public Health* 53 Suppl 1**,** 17-18.

Bett, B., Jost, C., Allport, R., and Mariner, J. (2009). Using participatory epidemiological techniques to estimate the relative incidence and impact on livelihoods of livestock diseases amongst nomadic pastoralists in Turkana South District, Kenya. *Prev Vet Med* 90(3-4)**,** 194-203.

Bonniwell, M.A. (1980). The use of tissue culture rinderpest vaccine (TCRV) to protect sheep and goats against "peste des petits ruminants" in the Ashanti region of Ghana. *Bulletin de l'Office International des Epizooties* 92**,** 1233-1238.

Couacy-Hymann, E., Bodjo, C., Danho, T., Libeau, G., and Diallo, A. (2005). Surveillance of wildlife as a tool for monitoring rinderpest and peste des petits ruminants in West Africa. *Rev Sci Tech* 24(3)**,** 869-877.

Diallo, A. (2000). "Peste des Petits Ruminants," in *OIE manual of standards for diagnostic tests and vaccines*. (Paris: Office International des Epizooties), 114-122.

Diallo, A. (2003). Control of peste des petits ruminants: classical and new generation vaccines. *Dev Biol (Basel)* 114**,** 113-119.

Drobne, S., and Lisec, A. (2009). Multi-attribute Decision Analysis in GIS: Weighted Linear Combination and Ordered Weighted Averaging. *Informatica* 33**,** 459-474.

FAO (1999). *Recognizing Peste des Petits Ruminants. A field manual.* Rome: Food and Agriculture Organization of the United Nations.

Furley, C.W., Taylor, W.P., and Obi, T.U. (1987). An outbreak of peste des petits ruminants in a zoological collection. *Vet Rec* 121(19)**,** 443-447.

Gopilo, A. (2005). *Epidemiology of Peste des Petits Ruminants virus in Ethiopia and molecular studies on virulence* Toulouse, France: Institut National Polytechnique de Toulouse.

Khan, H.A., Siddique, M., Sajjad ur, R., Abubakar, M., and Ashraf, M. (2008). The detection of antibody against peste des petits ruminants virus in sheep, goats, cattle and buffaloes. *Trop Anim Health Prod* 40(7)**,** 521-527.

Kinne, J., Kreutzer, R., Kreutzer, M., Wernery, U., and Wohlsein, P. (2010). Peste des petits ruminants in Arabian wildlife. *Epidemiol Infect* 138(8)**,** 1211-1214.

Lefevre, P.C., and Diallo, A. (1990). Peste des petits ruminants. *Rev Sci Tech* 9(4)**,** 935-981.

Lembo, T., Oura, C., Parida, S., Hoare, R., Frost, L., Fyumagwa, R., et al. (2013). Peste des petits ruminants infection among cattle and wildlife in northern Tanzania. *Emerg Infect Dis* 19(12)**,** 2037-2040. doi: 10.3201/eid1912.130973.

Lundervold, M., Milner-Gulland, E.J., O'Callaghan, C.J., Hamblin, C., Corteyn, A., and Macmillan, A.P. (2004). A serological survey of ruminant livestock in Kazakhstan during post-Soviet transitions in farming and disease control. *Acta Vet Scand* 45(3-4)**,** 211-224.

Martrenchar, A., Zoyem, N., Ngangnou, A., Bouchel, D., Ngo Tama, A.C., and Njoya, A. (1995). Etude des principaux agents infectieux intervenant dans l'étiologie des pneumopathies des petits ruminants au Nord-Cameroun (Study of the main infectious agents involved in the aetiology of pulmonary illness among small ruminants in Northern Cameroon.) *Rev Elev Med Vet Pays Trop* 48(2)**,** 133-137.

Munir, M. (2014). Role of wild small ruminants in the epidemiology of peste des petits ruminants. *Transbound Emerg Dis* 61(5)**,** 411-424. doi: 10.1111/tbed.12052.

Nanda, Y.P., Chatterjee, A., Purohit, A.K., Diallo, A., Innui, K., Sharma, R.N., et al. (1996). The isolation of peste des petits ruminants virus from northern India. *Vet Microbiol* 51(3-4)**,** 207-216.

Odo, B.I. (2003). Comparative study of some prevalent diseases of ecotype goats reaared in south eastern Nigeria. *Small ruminant research* 50**,** 203-207.

Osman, N.A., Ali, A.S., ME, A.R., and Fadol, M.A. (2009). Antibody seroprevalences against Peste des Petits Ruminants (PPR) virus in sheep and goats in Sudan. *Trop Anim Health Prod* 41(7)**,** 1449-1453.

Ozkul, A., Akca, Y., Alkan, F., Barrett, T., Karaoglu, T., Dagalp, S.B., et al. (2002). Prevalence, distribution, and host range of Peste des petits ruminants virus, Turkey. *Emerg Infect Dis* 8(7)**,** 708-712.

Rao, R.V. (2013). "Improved Multiple Attribute Decision Making Methods," in *Decision Making in Manufacturing Environment Using Graph Theory and Fuzzy Multiple Attribute Decision Making Methods,* ed. R.V. Rao. (London: Springer), 7-39.

Roeder, P.L., Abraham, G., Kenfe, G., and Barrett, T. (1994). Peste des petits ruminants in Ethiopian goats. *Trop Anim Health Prod* 26(2)**,** 69-73.

Saaty, T.L. (1977). A Scaling Method for Priorities in Hierarchical Structures. *J. Math. Psychology* 15**,** 234–281.

Shankar, H., Gupta, V.K., and Singh, N. (1998). Occurrence of peste des petits ruminants like diseases in small ruminants in Uttar Pradesh. *Indian J Anim Sci* 68(1)**,** 38–40.

Singh, R.P., Saravanan, P., Sreenivasa, B.P., Singh, R.K., and Bandyopadhyay, S.K. (2004). Prevalence and distribution of peste des petits ruminants virus infection in small ruminants in India. *Rev Sci Tech* 23(3)**,** 807-819.

Sow, A., Ouattara, L., Compaoré, Z., Doulkom, B.R., Paré, M., Poda, G., et al. (2008). Serologic prevalence of peste des petits ruminants in Soum Province, north Burkina Faso. *Rev Elev Med Vet Pays Trop* 1**,** 5-9.

Spiegel, K.A., and Havas, K.A. (2019). The socioeconomic factors surrounding the initial emergence of peste des petits ruminants in Kenya, Uganda, and Tanzania from 2006 through 2008. *Transbound Emerg Dis* 66(2)**,** 627-633. doi: 10.1111/tbed.13116.

Taylor, W.P. (1984). The distribution and epidemiology of peste des petits ruminants. *Preventive Veterinary Medicine* 2**,** 157-166.

Taylor, W.P., and Ali, Q. (2005). *Recognizing PPR. A field manual of Pakistan.* Government of Pakistan.

Tounkara, K., Traore, A., Sidibe, S., Samake, K., Diallo, B.O., and Diallo, A. (1996). Epidémiologie de la peste des petits ruminants (PPR) et de la peste bovine au Mali. Enquêtes sérologiques. *Rev Elev Med Vet Pays Trop* 49**,** 271-273.

Wang, Z., Bao, J., Wu, X., Liu, Y., Li, L., Liu, C., et al. (2009). Peste des petits ruminants virus in Tibet, China. *Emerg Infect Dis* 15(2)**,** 299-301.

Waret-Szkuta, A., Roger, F., Chavernac, D., Yigezu, L., Libeau, G., Pfeiffer, D.U., et al. (2008). Peste des petits ruminants (PPR) in Ethiopia: analysis of a national serological survey. *BMC Vet Res* 4**,** 34. doi: 10.1186/1746-6148-4-34.
